# Domain-specific neuropsychological investigation of CAA with and without intracerebral haemorrhage

**DOI:** 10.1007/s00415-023-11977-8

**Published:** 2023-09-06

**Authors:** Edgar Chan, Guendalina B. Bonifacio, Corin Harrison, Gargi Banerjee, Jonathan G. Best, Benjamin Sacks, Nicola Harding, Maria del Rocio Hidalgo Mas, H. Rolf Jäger, Lisa Cipolotti, David J. Werring

**Affiliations:** 1 Department of Neuropsychology, National Hospital for Neurology and Neurosurgery, London, UK; 2 Department of Brain Repair and Rehabilitation, Stroke Research Centre, UCL Queen Square Institute of Neurology, London, UK; 3 Comprehensive Stroke Service, National Hospital for Neurology and Neurosurgery, Queen Square, London, UK; 4 Neuroradiological Academic Unit, Department of Brain Repair and Rehabilitation, UCL Queen Square Institute of Neurology, London, UK

**Keywords:** Stroke, Cognition, Neuropsychology, Cerebral amyloid angiopathy, Dementia

## Abstract

**Background:**

Cerebral amyloid angiopathy (CAA) is associated with cognitive impairment, but the contributions of lobar intracerebral haemorrhage (ICH), underlying diffuse vasculopathy, and neurodegeneration, remain uncertain. We investigated the domain-specific neuropsychological profile of CAA with and without ICH, and their associations with structural neuroimaging features.

**Methods:**

Data were collected from patients with possible or probable CAA attending a specialist outpatient clinic. Patients completed standardised neuropsychological assessment covering seven domains. MRI scans were scored for markers of cerebral small vessel disease and neurodegeneration. Patients were grouped into those with and without a macro-haemorrhage (CAA-ICH and CAA-non-ICH).

**Results:**

We included 77 participants (mean age 72, 65% male). 26/32 (81%) CAA-non-ICH patients and 41/45 (91%) CAA-ICH patients were impaired in at least one cognitive domain. Verbal IQ and non-verbal IQ were the most frequently impaired, followed by executive functions and processing speed. We found no significant differences in the frequency of impairment across domains between the two groups. Medial temporal atrophy was the imaging feature most consistently associated with cognitive impairment (both overall and in individual domains) in both univariable and multivariable analyses.

**Discussion:**

Cognitive impairment is common in CAA, even in the absence of ICH, suggesting a key role for diffuse processes related to small vessel disease and/or neurodegeneration. Our findings indicate that neurodegeneration, possibly due to co-existing Alzheimer’s disease pathology, may be the most important contributor. The observation that general intelligence is the most frequently affected domain suggests that CAA has a generalised rather than focal cognitive impact.

**Supplementary Information:**

The online version contains supplementary material available at 10.1007/s00415-023-11977-8.

## Introduction

Cerebral amyloid angiopathy (CAA) is a small vessel disease characterised by the deposition of amyloid-β protein in the walls of small leptomeningeal and cortical blood vessels [1]. Traditionally, diagnosis of CAA most often comes after a symptomatic spontaneous lobar intracerebral haemorrhage (ICH), a core feature of the disease [2, 3]. However, increased recognition of the clinical profile and improved availability of neuroimaging has meant that identifying CAA prior to an acute ICH is becoming more common, enabling earlier management and treatment as well as an opportunity to differentiate possible effects of CAA pathology from the consequences of ICH. Established clinical and neuroimaging features of CAA include transient focal neurological episodes (TFNE), convexity subarachnoid haemorrhage, cortical microbleeds, superficial siderosis, white matter hyperintensities and perivascular spaces [4]. However, understanding of the specific impact of lobar ICH and more diffuse small vessel pathology on cognition remains incomplete. Such an understanding is important in allowing rational approaches to mitigate the cognitive impact of CAA.

In large-scale autopsy studies of community-based populations, CAA pathology is associated with worse cognitive impairment, increased likelihood of Alzheimer Disease (AD) and accelerated cognitive decline [5–8]. However, the nature of the cognitive difficulties implicated in the disease is still unclear. For example, while one study has suggested impairment in perceptual speed, episodic memory and semantic memory [6], others have found impairment in episodic but not semantic memory [7]. Some studies have argued, in fact, that memory is not severely affected in patients with CAA, but rather the profile of impairment is like that commonly found in vascular dementia with processing speed and executive functions being most affected [9, 10]. Moreover, some studies suggest that visuo-perceptual processing might be particularly affected in CAA due to the posterior cortical predilection of CAA pathology [11]; indeed, there is preliminary evidence that visuo-perceptual impairments correlate with white matter hyperintensities and other neuroimaging changes in posterior brain regions [12, 13]. A major limitation of these studies is that none have comprehensively assessed all the relevant domains of cognitive functioning simultaneously, thus making it difficult to draw accurate comparisons and potentially artificially emphasising certain domains. Previous studies reporting on cognitive performance on patients with CAA often combine patients with and without ICH into one group (e.g. [10, 13]), making it difficult to disentangle the contribution of CAA pathology alone.

The current study aimed to comprehensively define the domain-specific neuropsychological profile of patients with CAA and examine the association between commonly detected neuroimaging markers of cerebral small vessel disease and neurodegeneration with specific cognitive domains. We included patients with and without ICH to assess the potential contribution of lobar ICH and the more diffuse processes of the underlying vasculopathy, neurodegeneration or both.

## Methods

### Participants

Data were screened from a prospectively collected database of individuals who attended a specialist intracranial haemorrhage outpatient clinic at the National Hospital for Neurology and Neurosurgery (NHNN), Queen Square, London between Feb 2016 and Jan 2019. Inclusion criteria were as follows: (1) diagnosis of possible or probable CAA according to the modified Boston criteria [3], (2) completion of a comprehensive neuropsychological assessment and (3) an MRI within 28 days of the neuropsychological assessment (mean = 1 day). Exclusion criteria were as follows: (1) CAA-related inflammation, (2) ICH secondary to a non-CAA-related cause, (3) ischaemic stroke, (4) previous diagnosis of dementia and (5) any other co-morbid neurological condition or disease. Included patients were divided into two groups: those without ICH (CAA-non-ICH) and those with ICH (CAA-ICH). Patients in the CAA-non-ICH group had to have one or more of the following markers: (a) previous TFNE, (b) previous convexity subarachnoid haemorrhage (SAH) or (c) cortical superficial siderosis (cSS). Demographic information collected included age, sex, years of education and handedness.

### Neuropsychological assessment

All patients underwent a comprehensive neuropsychological assessment conducted by a Clinical Neuropsychologist, which included assessment of the following domains: verbal IQ, non-verbal IQ, verbal memory, non-verbal memory, language, visuo-perception, visuo-spatial function, executive functions, speed of information processing, anxiety and depression. Patients received a tailored collection of tests which was considered appropriate by the clinical neuropsychologist at the time (see Supplementary Information). Performance on tests was scored according to published standardised normative data. Impairment in verbal and non-verbal IQ was defined as a difference equal to or greater than 10 points between the patient’s actual performance and their estimated premorbid functioning based on the National Adult Reading Test (NART). Impairment in a focal cognitive domain was classed as scoring at or below the fifth percentile on any one test within the domain, except for the executive domain, where failure on two or more tests was required or only one if it was a screening measure (see Supplementary Information). Mood was classified as impaired if scores were ≥ 5 on the Geriatric Depression Scale (GDS) and/or if scores were within the ‘moderate’ to ‘severe’ range on the Hospital Anxiety and Depression Scale (HADS) or the Depression Anxiety Stress Scale (DASS-21) according to published cut-offs.

### Neuroimaging

MRIs were reviewed by GBB and BS under the supervision of HRJ and DJW. The presence and number of cerebral microbleeds (CMBs) were classified according to the Microbleed Anatomical Rating Scale (MARS) [14] on susceptibility-weighted imaging (SWI) MRI sequences. White Matter Hyperintensities (WMHs) were rated on T2-weighted sequences using the Fazekas scale for periventricular and deep WMH [15, 16]. Lacunes were identified and counted in accordance with standardised definition [17]. Enlarged Perivascular Spaces (EPVS) were rated on axial T2-weighted MRI using a validated 4-point visual rating scale (0 = no PVS, 1 = < 10 PVS, 2 = 11–20 PVS, 3 = 21–40 PVS and 4 = > 40 PVS) in the Basal Ganglia (BG) and Centrum Semiovale (CSO) [18]. cSS was classified on SWI sequences as ‘focal’ (involving 3 or less sulci) or ‘disseminated’ (4 or more sulci) [19]. Medial temporal atrophy (MTA) was rated on coronal FLAIR [20] using the Scheltens visual scale [21] and global cortical atrophy (GCA) was rated using the Pasquier scale on axial T2 inverted images [22].

### Statistical analyses

All data were analysed using SPSS Version 26.0 (IBM Corp). Data were analysed for skewness and kurtosis and tested for normality using the Kolmogorov–Smirnoff test. Independent *t* test and Mann–Whitney *U* test was used for normally and not-normally distributed continuous data, respectively. Chi-square or Fisher’s exact test was used to compare categorical variables. Cognitive profile of the non-ICH and ICH group was described using means (SD) for continuous variables and frequencies for categorical variables. Univariate and adjusted logistic regression analyses were used to examine the association between the individual neuroimaging markers and cognitive impairment (i.e. no impairment vs. impairment in any one or more domains). Significant variables (*p* < 0.1) were subjected to further univariate and adjusted logistic regression to examine its association with impairment in each individual cognitive domain (impaired or not). In adjusted analyses, age and ICH (those with vs. without macro-haemorrhage) were added as covariates. Age was included due to its well-documented impact on cognition, in both healthy ageing and in neurological patients [23]. ICH was included as we wanted to ensure that significant findings were not confounded by the possible impact of macro-haemorrhage, only applicable to approximately half the sample. As this analysis was exploratory, we did not adjust for multiple comparisons.

## Results

Seventy-seven patients were included in this study with 41.5% (*n* = 32) in the CAA-non-ICH group. The demographic and neuroimaging profile of the two groups are described in Table 1. Compared to patients with CAA-ICH, patients in the CAA-non-ICH group were significantly older (mean age 74.2 vs 70.2) and were more likely to have had TFNE (81.3% vs 22.2%), cSAH (75.0% vs 13.04%) and disseminated cSS (75% vs 44.4%), but less periventricular and deep WMH.

**Table 1:** Demographic and neuroimaging characteristics of patients with CAA-non-ICH and CAA-ICH

	Non-ICH	ICH	Total	*p* value
*n*	32	45	77	–
Age, in years, mean (SD)	74.2 (7.2)	70.2 (8.1)	71.8 (7.9)	**0.029**
Sex, male (%)	18 (56.3)	32 (71.1)	50 (64.9)	0.178
Years of education, mean (SD)	12.0 (2.5)	12.7 (3.1)	12.4 (2.9)	0.354
Handedness, number RH (%)	29 (93.5)	43 (95.6)	72 (94.7)	1
Previous TFNE, number (%)	26 (81.3)	10 (22.2)	36 (46.8)	** < 0.001**
Previous convexity SAH, number (%)	24 (75.0)	6 (13.3)	30 (39.0)	** < 0.001**
cSS criteria, none/focal/disseminated	1/7/24	11/13/20	12/20/44	**0.004**
Lobar CMBs (0, 1, 2–4, > 4)	3/2/7/20	2/1/6/35	5/3/13/55	0.398
Deep CMBs (0, 1, 2–4, > 4)	28/1/2/1	41/2/0/1	69/3/2/2	0.482
Fazekas periventricular scale (0/1/2/3)	3/13/8/8	0/13/13/19	3/26/21/27	**0.033**
Fazekas deep white matter scale (0/1/2/3)	1/22/6/3	1/21/13/10	2/43/19/13	**0.043**
Lacune number, mean (SD)	0.31 (0.59)	0.37 (0.68)	0.35 (0.64)	0.788
BGPVS rating (1/2/3/4)	19/10/1/2	22/18/3/2	41/28/4/4	0.409
CSOPVS rating (1/2/3/4)	4/5/10/13	5/9/13/18	9/14/23/31	0.909
Global cortical atrophy (0/1/2/3)	5/21/5/1	14/20/10/1	19/41/15/2	0.506
MTA (0/1/2/3/4)	12/9/10/0/1	12/16/10/5/2	24/25/20/5/3	0.310

Significant differences between CAA-non-ICH and CAA-ICH group (*p* < 0.1) are in bold

### Cognitive profile of CAA-non-ICH

The median number of domains impaired in the CAA-non-ICH group was 1 (range 0–5), with 81.2% of patients impaired in at least one domain. The frequency of impairment across cognitive domains is described in Table 2. Non-verbal IQ (59.3%) and verbal IQ (50%) were the most commonly impaired domains, followed by executive functions (37.5%) and speed of processing (30%). Memory, language and perceptual problems were much less common by comparison. Mood disorders were common, with anxiety (36.4%) more frequent than depression (20.8%).

**Table 2:** Cognitive domain-specific comparisons between non-ICH and ICH patient groups

		Non-ICH	ICH	Total	*p* value
	*n*	32	45	77	–
**General intelligence**					
Verbal IQ, number impaired (%)	61	13 (50.0)	17 (48.6)	30 (49.2)	0.912
Non-verbal IQ, number impaired (%)	61	16 (59.3)	23 (67.6)	39 (63.9)	0.498
**Memory**					
Verbal memory, number impaired (%)	74	2 (6.7)	8 (18.2)	10 (13.5)	0.187
Non-verbal memory, number impaired (%)	72	3 (9.7)	11 (26.8)	14 (19.4)	**0.080**
**Language, number impaired (%)**	77	7 (21.9)	13 (28.9)	20 (26.0)	0.489
**Perceptual functions**					
Visuo-perceptual, number impaired (%)	75	2 (6.5)	7 (15.9)	9 (12.0)	0.292
Visuo-spatial, number impaired (%)	48	1 (5.6)	3 (10.0)	4 (8.3)	1
**Executive functions, number impaired (%)**	77	12 (37.5)	16 (35.6)	28 (36.4)	0.861
**Speed of processing, number impaired (%)**	74	9 (30.0)	19 (43.2)	38 (37.8)	0.251
**Mood**					
Anxiety, number impaired (%)	50	8 (36.4)	10 (35.7)	18 (36.0)	0.962
Depression, number impaired (%)	57	5 (20.8)	15 (45.5)	20 (35.1)	**0.054**

Significant differences between CAA-non-ICH and CAA-ICH group (*p* < 0.1) are in bold

### Cognitive profile of CAA-ICH

**Fig. 1:**
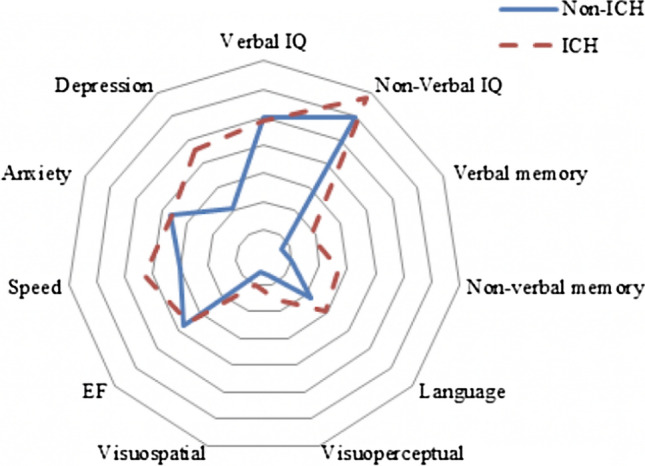
Comparison of the proportion of patients impaired in each domain split by non-ICH and ICH groups. Concentric lines represent increasing degree of impairment in 10% increments with outermost line representing 70%

The frequency and pattern of cognitive impairment across domains were similar between those with CAA-non-ICH and those with CAA-ICH (Fig. 1). The median number of domains impaired in the CAA-ICH group was 2 (range 0–6), which was higher than that of the CAA-non-ICH group, though there was no overall significant difference in the number of domains impaired between the groups (*p* = 0.173). Like the CAA-non-ICH group, non-verbal IQ (67.6%) and verbal IQ (48.5%) were again the most commonly impaired domains, followed by speed of processing (43.2%) and executive functions (35.6%). Memory, language and perceptual problems were more frequent than that in the CAA-non-ICH group, but the differences were not statistically significant (*p* > 0.05). In contrast to the CAA-non-ICH group, depression (45.5%) was more common than anxiety (35.7%).

To ensure that the pattern of results was not unduly influenced by patients who had severe atrophy which might be indicative of significant neurodegeneration, we re-ran the analysis removing patients with MTA/GCA scores ≥ 3; this did not change the pattern of results.

### Relationship between clinical and neuroimaging features and cognitive impairment.

We investigated which, if any, clinical and neuroimaging features of CAA and small vessel disease (SVD) were predictors of CAA cognitive impairment. First, we ran independent univariate logistic regression analyses to investigate the association of each neuroimaging feature with the likelihood of cognitive impairment (i.e. no impairment vs. impairment in any one or more domain). Previous TFNE, WMH and MTA were all found to be significant predictors (Table 3) and remained significant when we adjusted for age and ICH. None of the other clinical or neuroimaging features were significant, including lobar cerebral microbleeds and cSS.

**Table 3:** Logistic regression models (univariable and adjusted) examining the association between clinical and structural markers of CAA and SVD and the presence of cognitive impairment

	Univariable OR (95% CI)	*p* value	Adjusted OR (95% CI)	*p* value
Previous TFNE	5.57 (1.10–28.26)	**0.038**	5.70 (0.88–36.98)	**0.068**
Previous convexity SAH	1.05 (0.27–4.09)	0.942		
cSS (presence)	1.96 (0.23–17.00)	0.540		
Lobar CMBs (0, 1, 2–4, > 4)	0.96 (0.44–2.07)	0.913		
Deep CMBs (0, 1, 2–4, > 4)	0.71 (0.29–1.76)	0.463		
WMH (Fazekas periventricular 3 or Fazekas deep ≥ 2)	8.74 (1.05–72.83)	**0.045**	7.47 (0.86–64.73)	**0.068**
Lacune (presence)	1.47 (0.29–7.58)	0.646		
BGPVS rating (1/2/3/4)	1.63 (0.58–4.60)	0.355		
CSOPVS rating (1/2/3/4)	0.88 (0.45–1.72)	0.709		
Global cortical atrophy (0/1/2/3)	2.01 (0.74–5.51)	0.174		
MTA (0/1/2/3/4)	2.69 (1.06–6.79)	**0.037**	2.87 (1.08–7.57)	**0.034**

Each neuroimaging marker was considered independently. The adjusted model included age and ICH as covariate. *p* values < 0.1 are in bold

We then examined in more detail whether these three variables predicted impairment in each specific cognitive domain (Table 4). In univariate analysis, TFNE was a significant predictor of non-verbal memory impairment, but this was no longer significant in adjusted analysis. WMH was a significant predictor of non-verbal IQ and remained so in adjusted analysis. MTA was a significant predictor of verbal IQ, non-verbal IQ, language, visuo-spatial processing, executive functions and speed of processing. In adjusted analyses, MTA remained a predictor for all the aforementioned cognitive domains except visuo-spatial processing but was not a significant predictor of either verbal or non-verbal memory impairment.

**Table 4:** Logistic regression models (univariable, and adjusted (in italics) where applicable) examining the association between presence of TFNE, WMH and severity of MTA, and domain-specific cognitive impairment

Cognitive domain	TFNE	*p* value	WMH	*p* value	MTA	*p* value
	Univariable OR (95% CI) *Adjusted OR (95% CI)*		Univariable OR (95% CI) *Adjusted OR (95% CI)*		Univariable OR (95% CI) *Adjusted OR (95% CI)*	
Verbal IQ	1.22 (0.45–3.33)	0.699	2.08 (0.74–5.81)	0.163	2.56 (1.33–4.94) *3.07 (1.48–6.41)*	**0.005** ***0.003***
Non-verbal IQ	1.69 (0.59–4.85)	0.334	4.40 (1.35–14.33) *4.21 (1.23–14.34)*	**0.014** ***0.022***	1.97 (1.04–3.74) *2.41 (1.17–4.95)*	**0.037** ***0.017***
Verbal memory	2.33 (0.55–9.83)	0.248	1.29(0.34–4.88)	0.712	1.37 (0.75–2.48)	0.303
Non-verbal memory	4.84 (1.22–19.21) *3.71 (0.73–18.95)*	**0.025** *0.115*	2.18 (0.67–7.13)	0.197	0.89 (0.49–1.60)	0.690
Language	1.92 (0.67–5.53)	0.224	1.05 (0.38–2.92)	0.930	1.80 (1.10–2.95) *1.79 (1.09–2.94)*	**0.019** ***0.022***
Visuo-perceptual	2.00 (0.46–8.68)	0.355	1.81 (0.44–7.35)	0.409	1.53 (0.84–2.82)	0.165
Visuo-spatial	2.28 (0.22–23.68)	0.490	1.59 (0.20–12.36)	0.659	2.85 (0.98–8.29) *2.56 (0.66–9.93)*	**0.054** *0.175*
Executive functions	1.28 (0.50–3.26)	0.605	1.81 (0.71–4.66)	0.211	1.91 (1.18–3.08) *1.94 (1.18–3.19)*	**0.008** ***0.009***
Speed of processing	1.55 (0.60–4.01)	0.371	1.42 (0.55–3.66)	0.466	1.64 (1.02–2.63) *1.53 (0.94–2.50)*	**0.040** ***0.088***

Each clinical or neuroimaging marker was considered independently. The adjusted model included age and ICH as covariate. *p* values < 0.1 are in bold

## Discussion

To the best of our knowledge, this is the first study to comprehensively assess the domain-specific neuropsychological profile of patients with CAA and examine its association with commonly detected clinical and neuroimaging markers. Our main finding was that cognitive impairment is common in CAA even in the absence of ICH, with general intellectual functioning, executive functions and speed of processing being the most frequently affected domains.

Over 80% of the patients diagnosed with possible or probable CAA who had not suffered ICH were found to have impairment in at least one cognitive domain on neuropsychological testing. Notably, impairment in our study was classified as performing at or below the fifth percentile compared to normative data and thus represents a substantial deterioration in cognitive abilities. The frequency of impairment was similar in a previous study which combined both patients with and without ICH, but had a higher threshold for impairment (79% below the 16th percentile; [10]). In our study, patients with CAA who had a previous macro-haemorrhage were found to have a higher median number of cognitive domains impaired, which is not surprising given the known impact of cortical macro-haemorrhages on cognitive functioning [24, 25]. Interestingly, however, the pattern of impairment was strikingly similar between the two groups. For both groups, impairment in general intellectual functioning, executive functioning and speed of processing were the most frequent by far. Although impairment in memory, language and visuo-perceptual/spatial functions were detected in our sample, this was much rarer particularly in patients without a macro-haemorrhage. Our findings go against suggestions that CAA pathology is characterised by memory impairment [6] or visuo-perceptual/spatial impairment [12, 13]. The discrepancy in findings most likely reflects the fact that previous studies had limited cognitive batteries only assessing a few cognitive domains, therefore restricting the possibility to assess and compare across the full spectrum of cognitive functions. Furthermore, previous studies often considered patients with and without ICH in the same group, thereby likely conflating the results with the direct impact of symptomatic ICH rather than considering only the underlying CAA pathology or neurodegeneration per se.

Our finding that general intellectual functioning is the most commonly affected cognitive domain is a novel finding, not previously documented in the CAA literature. General intellectual functioning refers to the construct *g* which is thought to represent the common variance that is shared across diverse cognitive domains [26]. It is most commonly assessed using the Wechsler Adult Intelligence Scale [27], a test designed to measure general intelligence by summing performance across a battery of subtests measuring different verbal and non-verbal abilities. Importantly, it is distinct from what is sometimes referred to in the literature as ‘global’ cognition derived from averaging scores across an assorted test battery or using cognitive screening measures such as the MMSE or MoCA that do not include measures of general intelligence. Intellectual functioning has significant predictive validity not only for education and occupational success but also for health, illness, and death [28]. Low intellectual functioning in early life is a significant predictor of later life dementia [29], and decline in intellectual functioning is an early sign of disease onset in neurodegenerative diseases such as AD [30] and Huntington’s disease [31]. Our finding that one in two patients with CAA has impairment in general intellectual functioning suggests that it may also have a strong predictive value in disease progression and warrants further investigation and more systematic evaluation in future studies.

Our finding that general intellectual functioning is the most affected cognitive domain suggests perhaps that CAA pathology might initially have a generalised, rather than focal, impact on brain functioning. There is a long-held view that cerebral small vessel disease and vascular cognitive impairment are characterised by frontal-executive and processing speed impairments [32, 33]. Indeed, we also found that executive functions and speed of processing impairments were common in our sample, albeit not as common as intellectual decline. More recently, however, a systematic review suggested that cognitive impairment related to small vessel disease is more heterogeneous and can affect any/all cognitive domains [34]. We postulate that perhaps small vessel diseases such as CAA may primarily compromise general intellectual abilities, and this may be in keeping with the pathophysiological process of small vessel disease which is thought to be widespread, causing disruption to structural and functional white matter networks [35]. In support of this, neuroimaging studies have shown that patients with CAA have decreased brain volume [9] and global efficiency of structural brain networks [11, 36], and this reduction is associated with cortical amyloid burden and cognitive performance. In this formulation, strategic anatomical locations of some lesions can also cause distinct patterns of impairment, but this would be much rarer.

With regard to the clinical and neuroimaging variables, previously reported TFNE, severity of WMH and MTA were all significant predictors of cognitive impairment. Notably, CMB and cSS were not significantly predictive. When examining more closely the relationship with individual cognitive domains, WMH severity was a significant predictor of impairment in non-verbal IQ and MTA severity was a significant predictor of impairment in verbal IQ, non-verbal IQ, language, executive functions and speed of processing. TFNE was not a significant predictor of any individual cognitive domains after controlling for age and previous ICH. Our finding that TFNE is predictive for the presence of cognitive impairment, but not any individual cognitive domain, suggests perhaps that TFNE may be a useful marker for disease severity. Indeed, a recent meta-analysis showed that patients presenting with CAA-associated TFNEs are at a higher risk of lobar ICH and death during a follow-up period of up to 2.5 years, after controlling for cSS and cSAH [37]. In our study, we also did not find any significant association between cognitive impairments and cSS and cSAH, thus again suggesting that TFNE might be an independent clinical marker for CAA and cognitive risk. Our finding that WMH severity was also predictive of cognitive impairment and particularly impairment in non-verbal IQ is in keeping with the well-established relationship between WMH and CAA in the literature [11]. It has been shown, for example, that WMH progresses rapidly over time in CAA, particularly in those patients with cognitive impairment, and progression is associated with incident lobar haemorrhages [38]. Also, WMH damage on admission is related to cognitive impairment prior to an index ICH (e.g., [39]).

Our finding that MTA severity was also strongly associated with impairments in multiple cognitive domains is very striking. MTA is most commonly associated with Alzheimer’s pathology, and thus, it raises the possibility that perhaps there was an overlap of AD in our sample. Indeed, a recent study showed that memory impairment in CAA patients with elevated amyloid-PET retention was also associated with increased tau-PET binding and reduced hippocampal volume and the authors suggested that these patients likely have concomitant Tau pathology [40]. Interestingly, these patients also had worse cognitive performance in executive functions and language. Thus, more severe cognitive impairment may be an indicator of underlying presence of mixed pathology. However, it is unlikely that the cognitive impairment found in this study can be fully accounted for by AD pathology alone. Several points argue against this possibility. Firstly, patients with a previous clinical diagnosis of dementia were excluded from this study. Secondly, the pattern of cognitive impairment found is inconsistent with what is typically expected with AD pathology, namely, with memory as the primary early deficit; although MTA was a significant predictor of several cognitive domains, it was not significantly associated with memory impairment in our adjusted regression analysis. Thirdly, although we are unable to verify this in our study, the independent contribution of CAA to cognitive impairment separate from AD pathology has already been shown in previous studies [5–8]. Finally, it may be that MTA might also be a direct consequence of CAA pathology alone. The highest prevalence of CAA is often found in the occipital lobe in comparison with other neocortical regions [7, 8], which is served by the posterior circulation which also involves the hippocampus. Indeed, CAA pathology has been found in the hippocampus, and interestingly, in analyses adjusted for AD pathology, hippocampal CAA was not associated with episodic memory impairment [7].

Finally, mood disorders were common in patients with CAA. While anxiety (36.4%) was more frequent than depression (20.8%) in patients without a macro-haemorrhage, depression (45.5%) was more common than anxiety (35.7%) in patients who had a previous macro-haemorrhage. These rates are similar to those found in large-scale meta-analyses of mood disorders following stroke [41, 42]. Our findings highlight that screening and treating mood disorders in CAA needs to be a crucial part of the clinical care for patients with CAA, even in those who have not had a macro-haemorrhage. Mood difficulties not only have an impact on quality of life and well-being but can also have a direct impact on cognitive functioning and increased risk for cardiovascular disease and mortality [43, 44].

Some limitations need to be considered. Due to the relatively small sample size of the study relative to the number of variables, we had to dichotomize cognitive impairment as either impaired or not impaired. As such, we were unable to assess whether the severity of impairment differed across domains. This may provide further valuable information about the nature of cognitive difficulties that result from CAA. The relatively small sample size also means we could be underpowered to detect more subtle differences between groups. We also did not have PET or CSF data for our sample, so we could not clarify the potential overlap between CAA and AD pathology. Finally, our patient sample was drawn from those who attended a specialist CAA clinic; thus, it may not be representative of a community sample or perhaps those who attended a specialist memory clinic.

In conclusion, our comprehensive neuropsychological investigation showed that cognitive impairment is common in CAA, even in the absence of a macro-haemorrhage. Neuropsychological assessment should be a standard part of clinical care of patients with suspected or confirmed CAA, particularly in those with reported TFNE and/or neuroimaging features of WMH and MTA. Further work is needed to examine the evolution of cognitive impairment in CAA over time, and the interaction between CAA and AD pathology.

### Supplementary Information

Below is the link to the electronic supplementary material.

Supplementary file1 (DOCX 19 KB)
