# Supplementary material for: Domain-specific neuropsychological investigation of CAA with and without intracerebral haemorrhage
Source: J Neurol. 2023 Sep 6;270(12):6124–32. doi: 10.1007/s00415-023-11977-8 (PMC10632296; doi:10.1007/s00415-023-11977-8)
Supplement: Supplementary file 1 — Supplementary file1 (DOCX 19 KB) [file 415_2023_11977_MOESM1_ESM.docx]

**Supplementary Information**

**List of Neuropsychological Tests Administered**

| **Premorbid Intellectual functioning** |
| --- |
| National Adult Reading Test (NART) [1] |
| Schonell Graded Word Reading Test [2] |
| **General intellectual functioning** |
| Wechsler Adult Intelligence Scale – 3^rd^ Edition (WAIS-III) [3] |
| Raven Advanced Progressive Matrices – Set 1 [4] |
| **Memory** |
| Recognition Memory Tests (RMT), Words and Faces [5] |
| Adult Memory and Information Processing Battery (AMIPB), Story and Figure recall [6] |
| The Camden Memory Test: Topographical Recognition Memory Test [7] |
| The Camden Memory Test: Paired Associate Learning Test [7] |
| The Doors and People Test [8] |
| **Naming** |
| Graded Naming Test [9] |
| Oldfield Naming Test [10] |
| **Visuo-perception** |
| Visual Object and Space Perception Battery (VOSP) [11] |
| **Executive functions** |
| Stroop Colour Word Test [12] |
| Hayling Sentence Completion Test [13] |
| Modified Card Sorting Test [14] |
| Cognitive Estimation Test [15] |
| Phonemic fluency [16] |
| **Speed of Processing** |
| Symbol Digit Modalities Test (SDMT) [17] |
| Trail-Making Test Part A [18] |
| ‘A’ Cancellation [19] |

**References**

1. Nelson, & Willison, J. (1991). National adult reading test (NART) : test manual / Hazel E. Nelson with Jonathan Willison (Part II). (2nd edition). NFER-Nelson.
2. Schonell, F. (1942). Backwards in the Basic Subjects. London: Oliver & Boyd.
3. Wechsler, D. (1997). WAIS-­‐III administration and scoring manual. The Psychological Corporation, San Antonio, TX
4. Raven, Raven, J. C., Court, J. H., Raven, J. C. (John C. ., & Court, J. H. (John H. (1992). Manual for Raven’s progressive matrices and vocabulary scales / by J. Raven, J.C. Raven and J.H. Court.H.K.Lewis & col. ltd.
5. Warrington E. (1984). Recognition Memory Test. Windsor: Nfer-Nelson.
6. Coughlan AK, Hollows SE. The adult memory and information processing battery: The manual. Leeds: Coughlan, 1985.
7. Warrington. (1996). The Camden memory tests / Elizabeth K. Warrington. Psychology Press.
8. Baddeley, A. D., Emslie, H., & Nimmo-Smith, I. (2006). Doors and people: A test of visual and verbal recall and recognition. Harcourt Assessment.
9. Warrington, E. K. (1997). The Graded Naming Test: A Restandardisation. Neuropsychological Rehabilitation, 7(2), 143–146. <http://doi.org/10.1080/713755528>
10. Oldfield RC, Wingfield A. Response latencies in naming objects. Q J Exp Psychol 1965;17:273–81.
11. Warrington EK, James M. Visual Object and Space Perception Battery. Bury St.
12. Trenerry, M. R., Crosson, B., DeBoe, J., & Leber, W. R. (1989). Stroop neuropsychological screening test. Odessa, FL: Psychological Assessment Resources.
13. Burgess, P. W., & Shallice, T. (1997). The hayling and brixton tests. Technical Report, Thames Valley Test Company, Bury St. Edmunds (UK), 1997
14. Nelson, H. E. (1976). A modified card sorting test sensitive to frontal lobe defects. Cortex, 12(4), 313-324.
15. MacPherson, Wagner, G. P., Murphy, P., Bozzali, M., Cipolotti, L., & Shallice, T. (2014). Bringing the cognitive estimation task into the 21st century: normative data on two new parallel forms. PloS One, 9(3), e92554–e92554. https://doi.org/10.1371/journal.pone.0092554
16. Tombaugh, Kozak, J., & Rees, L. (1999). Normative Data Stratified by Age and Education for Two Measures of Verbal Fluency: FAS and Animal Naming. Archives of Clinical Neuropsychology, 14(2), 167–177. https://doi.org/10.1016/S0887-6177(97)00095-4
17. Smith, A. (1982). Symbol digit modalities test (p. 22). Los Angeles, CA: Western Psychological Services.
18. Army Individual Test Battery (1944). Manual of directions and scoring. Washington, DC: War Department, Adjutant General’s Office.
19. Willison JR, Warrington EK. Cognitive retardation in a patient with preservation of psychomotor speed. Behav Neurol 1992;5:113–6.
